# Root system traits impact early fire blight susceptibility in apple *(Malus × domestica)*

**DOI:** 10.1186/s12870-019-2202-3

**Published:** 2019-12-23

**Authors:** Jugpreet Singh, Jack Fabrizio, Elsa Desnoues, Julliany Pereira Silva, Wolfgang Busch, Awais Khan

**Affiliations:** 1 Plant Pathology and Plant-Microbe Biology Section, Cornell University, Geneva, NY 14456 USA; 2 Salk Institute for Biological Studies, Plant Molecular and Cellular Biology Laboratory, and Integrative Biology Laboratory, 10010 N Torrey Pines Rd, La Jolla, CA 92037 USA

**Keywords:** *Erwinia amylovora*, Disease resistance, Root growth, Root mass, Root shoot interactions, Gene expression, Transcriptome, Co-expression network, Gene regulation, Grafting

## Abstract

**Background:**

Although it is known that resistant rootstocks facilitate management of fire blight disease, incited by *Erwinia amylovora,* the role of rootstock root traits in providing systemic defense against *E. amylovora* is unclear. In this study, the hypothesis that rootstocks of higher root vigor provide higher tolerance to fire blight infection in apples is tested. Several apple scion genotypes grafted onto a single rootstock genotype and non-grafted ‘M.7’ rootstocks of varying root vigor are used to assess phenotypic and molecular relationships between root traits of rootstocks and fire blight susceptibility of apple scion cultivars.

**Results:**

It is observed that different root traits display significant (*p* < 0.05) negative correlations with fire blight susceptibility. In fact, root surface area partially dictates differential levels of fire blight susceptibility of ‘M.7’ rootstocks. Furthermore, contrasting changes in gene expression patterns of diverse molecular pathways accompany observed differences in levels of root-driven fire blight susceptibility. It is noted that a singular co-expression gene network consisting of genes from defense, carbohydrate metabolism, protein kinase activity, oxidation-reduction, and stress response pathways modulates root-dependent fire blight susceptibility in apple. In particular, WRKY75 and UDP-glycotransferase are singled-out as hub genes deserving of further detailed analysis.

**Conclusions:**

It is proposed that low root mass may incite resource-limiting conditions to activate carbohydrate metabolic pathways, which reciprocally interact with plant immune system genes to elicit differential levels of fire blight susceptibility.

## Background

Roots play critical roles in plant function and their interactions with biotic and physical environments. Plant roots are increasingly recognized for their role in modulating systemic defenses of plants against pathogen infections via inter-organ signaling [1–4]. Roots can trigger physiological and genetic responses leading to activation of molecular pathways to recognize and resist pathogens upon infection [2–5]. Indeed, some root traits can act as physical barriers to soil-borne pathogens by hindering their penetration into living tissues [6–9]. Therefore, investigating interactions between roots and pathogens, as well as their relationships to disease susceptibility is of particular relevance to fruit tree crops wherein specific rootstocks are frequently chosen to confer disease resistance for susceptible scion cultivars [10, 11].

It has been reported that root system architecture (RSA) is dictated by growth, length, diameter, density, branching pattern, and branching angle of various root types, and it influences resource uptake from the soil [12–15]. In general, plant roots consist mainly of either one or more primary roots that, in-turn, produce several secondary and tertiary roots [15, 16]. In contrast, the root system of apple rootstocks in commercial orchards consists mainly of adventitious roots originating from nodal junctions of stem cuttings, via vegetative propagation, which are important for initial establishment and success of grafted scions. Thus, growth and density of adventitious roots can influence the nutrient acquisition capacity of a plant, both under normal and stress prone conditions [17]. Nutrient uptake not only supports overall plant growth, but also contributes to plant survival under different stress conditions, such as wounding, flooding, drought, and nutrient deficiency [17–19]. However, the potential role of adventitious roots in enhancing tolerance to biotic and abiotic stresses remains unclear.

Rootstocks impact scion genotypes in many different ways. They can influence scion vigor and architecture, phenology, precocity, fruit quality, and production [20, 21]. In addition, rootstocks confer differential tolerance to salinity, drought, and disease-prone conditions in various crops [21–23]. For example, resistant rootstocks have been selected and used to enhance disease tolerance of grafted scion cultivars [11, 24–26] for sustainable disease management in commercial apple orchards. It has been proposed that rootstocks can modify scion phenotypes by altering levels of abscisic acid, cytokinin, auxin, and other hormones through long-distance signaling between roots and shoots [27–30]. Moreover, rootstock-regulated gene expression differences and mobile mRNA movements may also contribute towards enhanced host defense against pathogen infection [11, 25, 31]. For example, rootstocks influence expression levels of disease-associated genes of jasmonic acid and inositol pathways in grafted apple scions under fire blight infection [11]. These rootstock-derived mobile mRNAs may act as long-distance signals [31] to alter expression of disease-related molecular pathways in grafted scions. Roots also produce secondary metabolites, such as nicotine, furocoumarins, and aldehydes to improve plant defense mechanisms against pathogens [4]. In contrast, foliar bacterial infection alters secretion of malic acid in roots to recruit beneficial soil bacteria and improve plant immunity against pathogen attack [32]. Furthermore, it is likely that rootstocks may also influence scion physiology by regulating levels of water and nutrient uptake [22, 33], which in turn can impose limits on pathogen spread and disease infection. Overall, root traits of rootstocks can play critical functional roles in regulating above-ground plant physiology and disease susceptibility of scions.

Fire blight, a systemic bacterial disease incited by *Erwinia amylovora* (Burr.) [34] causes extensive apple production losses worldwide. Fire blight infection can occur at multiple stages of plant development with higher risks of infection occurring particularly in new growing tissues of young orchards [35, 36]. Apple growers mainly rely on use of chemical treatments and of pruning of infected twigs to control fire blight in commercial orchards, but these preventive control measures remain inefficient once bacteria have already invaded reproductive and/or vegetative plant tissues. Plant resistance provides alternative options for sustainable control of bacterial spread, particularly once bacteria penetrate host tissues.

Use of resistant rootstocks serves to directly manage fire blight infection of rootstocks, but it can also limit its spread to susceptible scions [26, 36]. For example, susceptible scion cultivars grafted onto G.16, G.30, and G.11 apple rootstocks from the Geneva, New York apple rootstock breeding program have demonstrated high to moderate levels of resistance against fire blight [26, 37]. This observed rootstock-driven differential fire blight resistance of grafted scions is attributed to changes in gene expression of disease-related proteins and pathways, including those of phytohormones, transcription and signal transduction activities, as well as of various cellular and metabolic responses [11]. Rootstocks can potentially utilize several mechanisms to confer resistance to scions, but the precise mechanism of rootstock-defined scion resistance or tolerance to fire blight remains unknown.

In this study, we have tested the hypothesis that apple rootstocks of higher root mass (g) can respond more effectively to fire blight infection. To pursue this, the following two experiments have been conducted. In one experiment, a range of apple scion genotypes are grafted onto a single apple rootstock, ‘Malling 7’ (‘M.7’), grafted trees are allowed to grow, and are then challenged with artificial inoculation with *E. amylovora* to establish the relationship between root vigor and disease severity in variable genetic backgrounds*.* In the second experiment, non-grafted ‘M.7’ rootstocks of varying root mass are grown, and then these are challenged with *E. amylovora* to specifically test the effect of root vigor on disease severity in a single genotype. Morphological characteristics were evaluated in both the experiments that led to assess molecular interactions between root traits of rootstock and fire blight susceptibility of apples.

## Results

### Rootstock root traits and fire blight susceptibility of grafted scions are correlated

Data from 45 grafted scions on M.7 rootstocks (Additional file 9: Table S1) were used to evaluate the relationships between root, shoot, leaf, and fire blight infection severity traits. Both root dry mass (g) and average roots per node (count) showed a wide range of variation in this population of scion genotypes. The root dry mass of M.7 rootstocks varied from 0.67 g (g) to 6.84 g (Additional file 1: Figure S1A), whereas average number of roots per node ranged from 2.96 to 7.56 in M.7 rootstocks (Additional file 1: Figure S1B). Furthermore, corresponding shoot and leaf traits also showed significant (*p* < 0.05) variations in this population (Additional file 1: Figure S1C-E). Specifically, shoot and leaf lengths varied from 8.8 to 24.5 cm and 3.0 to 6.6 cm in this population, respectively, while SPAD values for leaf chlorophyll measurements ranged from 28.4 to 38.5. Moreover, shoot traits showed moderate broad-sense heritability (H^2^) values, ranging from 0.70 to 0.62, for leaf length and for leaf chlorophyll content. Of particular interest, percent lesion length ranged from 1.2 to 93% and showed significant (*p* < 0.05) variation in the population (Additional file 1: Figure S1F). The H^2^ values for percent lesion length was 0.71.

**Fig. 1:**
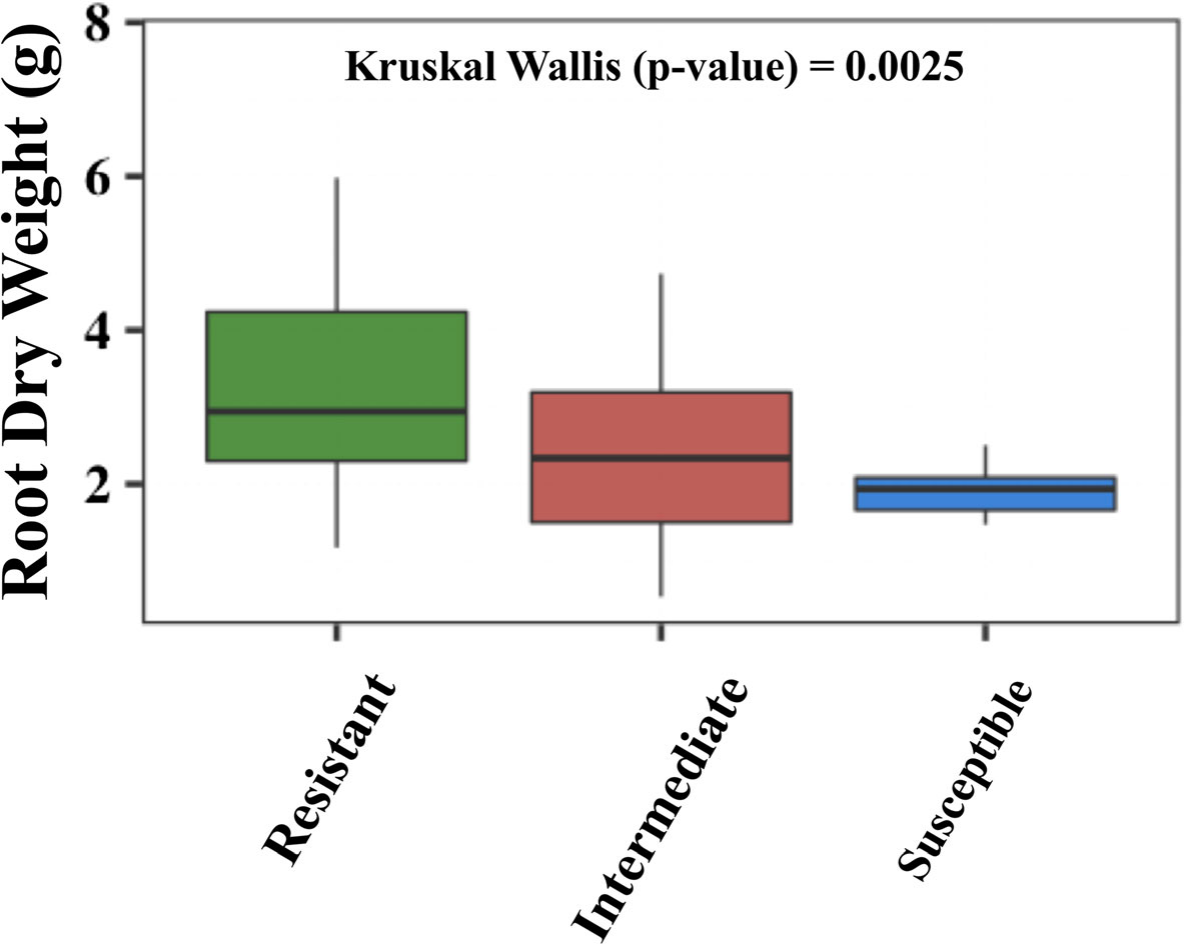
Box plot showing distribution of root dry mass (g) in three disease severity classes observed in 45 grafted scion genotypes on ‘M.7’ rootstocks. The percent lesion length classes were defined as Resistant (0–20% average PLL), Intermediate (21–80% average PLL), and Susceptible (81–100% average PLL). Error bars correspond to standard deviations of means

To specifically examine the relationships between root dry mass and fire blight percent lesion length, we performed a statistical analysis of root dry mass (g) by dividing the 45 scion genotypes into three classes based on percent lesion length (Fig. 1). The clustering of scion genotypes led to increased numbers of replications per disease severity class for more robust statistical analysis. A significant (*p* < 0.05) difference was observed in the root dry mass (g) between three disease severity classes (Fig. 1). Similar results were obtained after removing the 20 scion genotypes displaying high standard deviation in percent lesion length (Additional file 9: Table S1).

Pairwise phenotypic correlations showed positive correlations between root and shoot traits, and negative correlations between root and fire blight susceptibility traits (Additional file 10: Table S2). However, not all correlations were significant. For instance, root dry mass (g) had significant (*p* < 0.05) negative correlation of − 0.45 with percent lesion length (Additional file 2: Figure S2), whereas negative correlations between average roots per nodes and fire blight susceptibility traits were not significant. Similarly, root dry mass (g) displayed significant (*p* < 0.05) positive correlations with leaf length, but not with shoot length (Additional file 10: Table S2). Overall, these phenotypic correlations suggested that root dry mass of the rootstock could influence leaf growth and fire blight susceptibility of grafted scions.

**Fig. 2:**
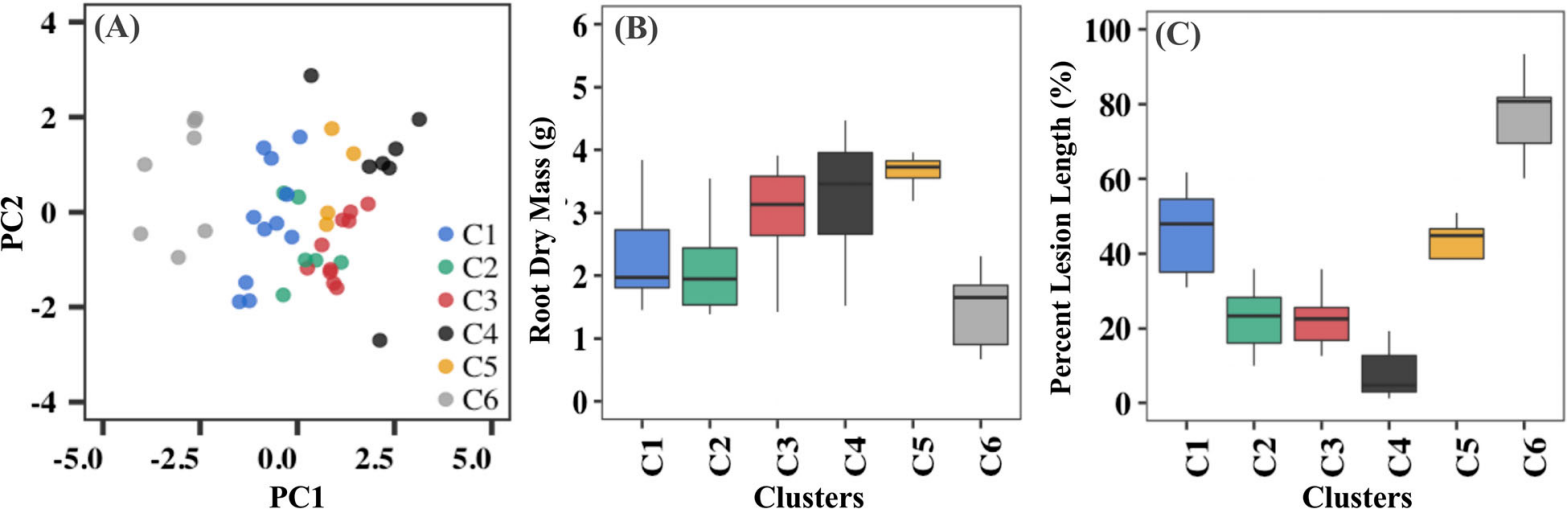
Genotype clustering based on principal component analysis (PCA) and hierarchical clustering of root and disease traits. (**a**) Six genotype clusters obtained from a population of 45 scion genotypes grafted onto ‘M7’ rootstocks. Distribution of means and variances in each cluster for (**b**) root dry mass (g) for C1–2.3, C2–2.2, C3–3.0, C4–3.8, C5–3.7, and C6–1.5; (**c**) percent lesion length (%) for C1–45.9, C2–22.6, C3–22.4, C4–9.4, C5–40.5, and C6–76.7

Hierarchical clustering and multivariate analysis were used to categorize the entire population into groups based on their phenotypic differences. Therefore, this population was divided into six main clusters exhibiting distinct trait variation patterns, as illustrated in the heat map of traits values (Additional file 3: Figure S3). Furthermore, PCA of root and fire blight disease traits also highlighted phenotypic differences of these genotype clusters in this population (Fig. 2a). For example, root dry mass (g) and percent lesion length (%) showed variable distribution patterns of trait means among the six identified clusters (Fig. 2b and c). In fact, clusters “C4” and “C6” tended to consist of genotypes with relatively lower root mass (g) and higher disease susceptibility. However, this observed pattern was less clearly demonstrated in the remaining clusters.

Interestingly, nine principal components (PCs) explained the total variation present in this population. PC1 explained a maximum of 32.6% of the total variation. Although all traits contributed towards PC1 variation, root and shoot traits had positive contributions, while fire blight disease susceptibility traits contributed negatively to PC1 variation (Additional file 4: Figure S4). This trend supported previously detected negative correlations between root and fire blight susceptibility traits. In addition, analysis of higher-order PCs revealed different levels of contributions from root, shoot, and disease susceptibility traits (Additional file 4: Figure S4), as noted by positive contributions from all root and disease-related traits to the PC2 variation. Overall, the PCA analysis revealed presence of considerable variation in this population, and this was partially driven by identified correlations between root growth and disease susceptibility traits.

### Rootstocks exceeding a root area threshold are less susceptible to fire blight

**Fig. 3:**
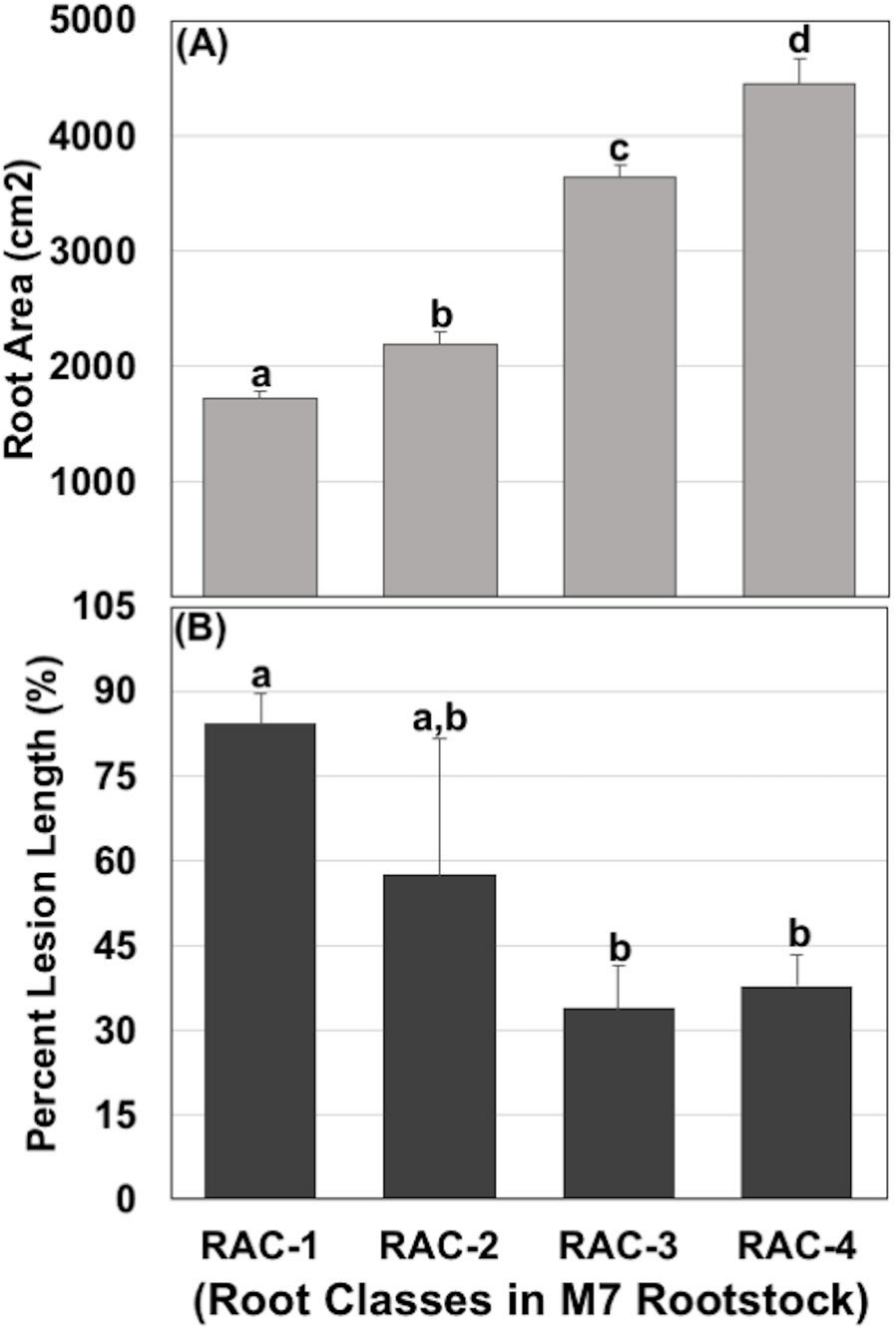
Patterns of root area (cm^2^) and disease severity (%) in four different root area classes (RACs) of ‘M.7’ rootstocks. (**a**) Different root area classes (RACs) observed in M.7 rootstock, and (**b**) Disease severity represented as percent lesion length (%) for four M.7 RACs at 8 dai. The different letters mean significant differences at a p-value threshold less than 0.05

To evaluate the extent to which roots can influence levels of fire blight susceptibility, a second independent experiment was conducted using non-grafted M.7 rootstocks representing four distinct root area classes (RACs). It was found that average root surface areas ranged from approximately 1720 (lowest RAC-1) to 4455 cm^2^ (highest RAC-4), corresponding to about 1.27 to 2.59-fold change between the lowest and the other three RACs (Fig. 3a). Moreover, fire blight infection, measured as percent lesion length (%), showed significant (*p < 0.05*) variations among the different RACs over time (Additional file 5: Figure S5). It was observed that absolute rates of disease progression from 2 to 8 dai were about 41.9 to 75.0% in RAC-4 and RAC-3; whereas, these were higher, 84.4 to 98.3%, in RAC-1 and RAC-2, respectively. At 8 dai, percent lesion length was significantly (*p* < 0.05) different in RAC-1 from those of RAC-3 and RAC-4, while this was not significantly different, at *p* < 0.05, in RAC-2 from those of the other RACs (Fig. 3b). Overall, total infection and progression of disease were comparatively less in root classes of high root surface areas (cm^2^) at the start of the experiment, and vice-versa. Moreover, the highest fire blight susceptibility was observed in rootstocks with a threshold of root surface area of 3644 cm^2^, represented by RAC-3 (Fig. 3).

Analysis of phenotypic correlations showed a strong correlation (r^2^ = 0.87; *p* < 0.05) of root areas (cm^2^) between pre- and post-planting (and following bacterial inoculation), for a total duration of 106 days of growth, thus indicating that initial root area could serve as a predictor of root area growth at later stages of root development. Similarly, other root traits also demonstrated significant (*p* < 0.05) positive correlations (Additional file 11: Table S3). For instance, root dry mass (g) showed high positive correlations with pre- and post-plant root area (r^2^ = 0.82 and 0.93, respectively). Similarly, both coarse and fine root mass showed significantly (*p* < 0.05) high positive correlations with root area (cm^2^) before (r^2^ = 0.79 and 0.62, respectively) and after planting (r^2^ = 0.85 and 0.78, respectively). Some root traits also displayed significant (*p* < 0.05) negative correlations with fire blight susceptibility traits. For instance, pre-plant root area (cm^2^) and fine root dry mass (g) had significant negative correlations of − 0.70 and − 0.58 with percent fire blight lesion length, respectively (Additional file 11: Table S3). In contrast, negative correlations of percent lesion length against post-planting root area (cm^2^), coarse root dry mass (g), and total dry mass (g) were not significant.

### Contrasting expression patterns of distinct sets of genes are associated with root-dependent fire blight susceptibility

**Fig. 4:**
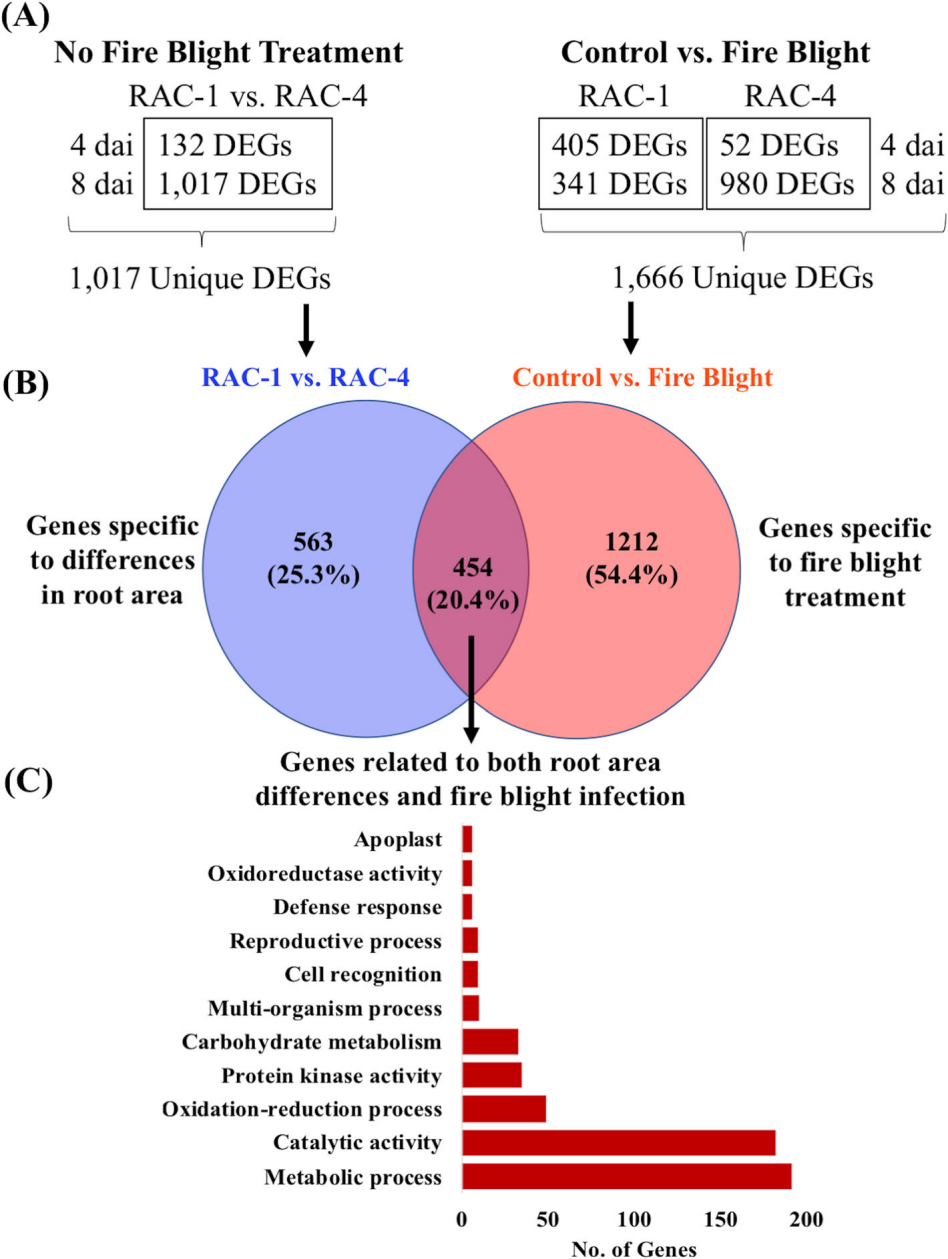
A schematic representation of differential gene expression and pathway analysis for root-dependent fire blight infection in apple. (**a**) Numbers of differentially expressed genes (DEGs) obtained from analysis of control samples between RAC-1 and RAC-4, and between control and fire blight samples within RAC-1 and RAC-4. The DEGs were identified with a log2fold change of 1.5 between control and fire blight treated samples that also exhibit p-value of less than 0.01, (**b**) Venn diagram with numbers of unique and shared DEGs from two different expression analyses. (**c**) Pathways showing overrepresentation from common DEGs that most likely correspond to effects of low root area and fire blight infection

Following bacterial inoculation, phenotypic analysis identified significant (*p < 0.05*) differences in fire blight infection over time in leaves of M.7 rootstocks belonging to different RACs (Fig. 3; Additional file 5: Figure S5). To identify molecular changes related to root-regulated fire blight susceptibility, gene expression patterns were characterized in leaf tissues of contrasting root area classes (RAC-1 with an average root area of 1720 cm^2^ vs. RAC-4 with an average root area of 4455 cm^2^) of non-grafted M.7 genotypes under control and bacterial inoculation treatments, at 4 and 8 dai (Additional file 12: Table S4). This gene expression analysis was conducted in sequential steps (Fig. 4). We first compared control leaf samples between RAC-1 and RAC-4 to identify any differentially expressed genes (DEGs) accounting for effects of root surface area differences on leaf responses. Next, control and bacterial-inoculated leaf samples were analyzed over time to identify fire blight responsive genes in leaf tissues. As a result, a set of common genes from both analyses were identified and deemed as genes associated with root-regulated fire blight susceptibility responses in leaf tissues.

Subsequently, it was observed that a high PC1 variation (82%) was present in this population (Additional file 13), thus suggesting that root area, fire blight infection, and sampling time contributed to the variability detected in the gene expression dataset. Furthermore, the number of significant (*p < 0.05*) DEGs increased over time in both control and bacterial-inoculated RAC-4 samples, while these decreased in bacterial-inoculated RAC-1 samples over time (Fig. 4a). Interestingly, a total of 132 and 1017 DEGs were detected between RAC-1 and RAC-4 at 4 and 8 dpi, respectively (Table 1; Additional file 14). All 132 DEGs at 4 dpi were also identified at 8 dpi (Additional file 6: Figure S6), which indicated that the effects of root surface area differences persisted, and in fact they became more severe over this developmental period. In addition, a set of 454 out of 1017 DEGs showed significant (p < 0.05) changes in transcript levels following bacterial infection (Fig. 4b; Additional file 15). These DEGs involved genes likely related to bacterial infection, whose expression levels were also dependent on differences in root surface areas, as noted in contrasting RACs, and referred to herein as root-regulated fire blight responsive (RRFBR) genes.

**Table 1:** Total number and percentage of differentially expressed (DE) genes for each comparison between two root area classes (RACs); lowest (RAC-1) and highest (RAC-4), and between control and fire blight (FB) infected samples at two time points after infection. The percentage of DE genes was calculated by comparing against total expressed genes (*n* = 35,224) in the transcriptome dataset. The genes were defined as DE based on the *p*-value < 0.01 and log2Fold change of 1.5 from likelihood ratio test statistics using DESeq2. In the RAC-1 vs RAC-4 comparisons, the induced genes have comparatively higher gene expression in RAC-1, whereas repressed genes have comparatively higher gene expression in RAC-4

Treatment	Time	Comparison	No. of DE Genes	DE Genes (%)	Induced	Repressed
Root Area Classes	1	RAC-1 vs RAC-4	132	0.37	24	108
	2	RAC-1 vs RAC-4	1017	2.88	412	605
Fire Blight Infection	1	RAC-1 (Control vs FB Treatment)	405	1.15	377	28
	1	RAC-4 (Control vs FB Treatment)	52	0.15	39	13
	2	RAC-1 (Control vs FB Treatment)	341	0.97	240	101
	2	RAC-4 (Control vs FB Treatment)	980	2.78	549	431

An analysis of normalized expression for RRFBR genes identified opposite trends in contrasting RACs at both 4 and 8 dai. For instance, about 31.7% (*n* = 144) DEGs demonstrated increased expression levels in RAC-1, but decreased expression levels in RAC-4 following bacterial inoculation at both sampling times (Additional file 16). Likewise, 11.4% (*n* = 53) DEGs demonstrated decreased expression levels in RAC-1, but increased expression levels in RAC-4. Interestingly, only a few genes (*n* = 9) demonstrated similar patterns of changes in expression levels between two these RACs following bacterial infection (Additional file 16). These findings suggested that for the majority of RRFBR genes, differences in fire blight susceptibility between RAC-1 and RAC-4 were mostly associated with contrasting gene expression patterns.

### Interactions between genes from multiple pathways accompany root-dependent fire blight susceptibility

Upon further gene ontology (GO) analysis, it was noted that ~ 92% of RRFBR genes belonged to general stress response pathways related to metabolic response (42%), catalytic activity (40%), and oxidation-reduction (10%) processes (Fig. 4c; Additional file 17), whereas 8% of RRFBR genes represented functional terms related to carbohydrate metabolic process, cell recognition, response to biotic stimulus, protein serine/threonine kinase activity, and apoplast (Fig. 4c; Additional file 17). Moreover, expression levels of DEGs varied within these pathways (Additional file 7: Figure S7). For example, some DEGs in carbohydrate metabolic and protein kinase pathways had lower levels of expression in RAC-1 than in RAC-4, while other DEGs displayed an opposite trend (Additional file 7: Figure S7). Interestingly, all six DEGs involved in defense response pathways demonstrated reduced levels of expression in RAC-1, but increased levels of expression in RAC-4 at 8 dai following bacterial infection (Additional file 7: Figure S7; Additional file 17). Overall, these findings pointed toward likely interactions of both general stress response and carbohydrate metabolism pathways with defense-related genes. Indeed, these interactions would explain root-regulated differences in fire blight susceptibility in apple.

**Fig. 5:**
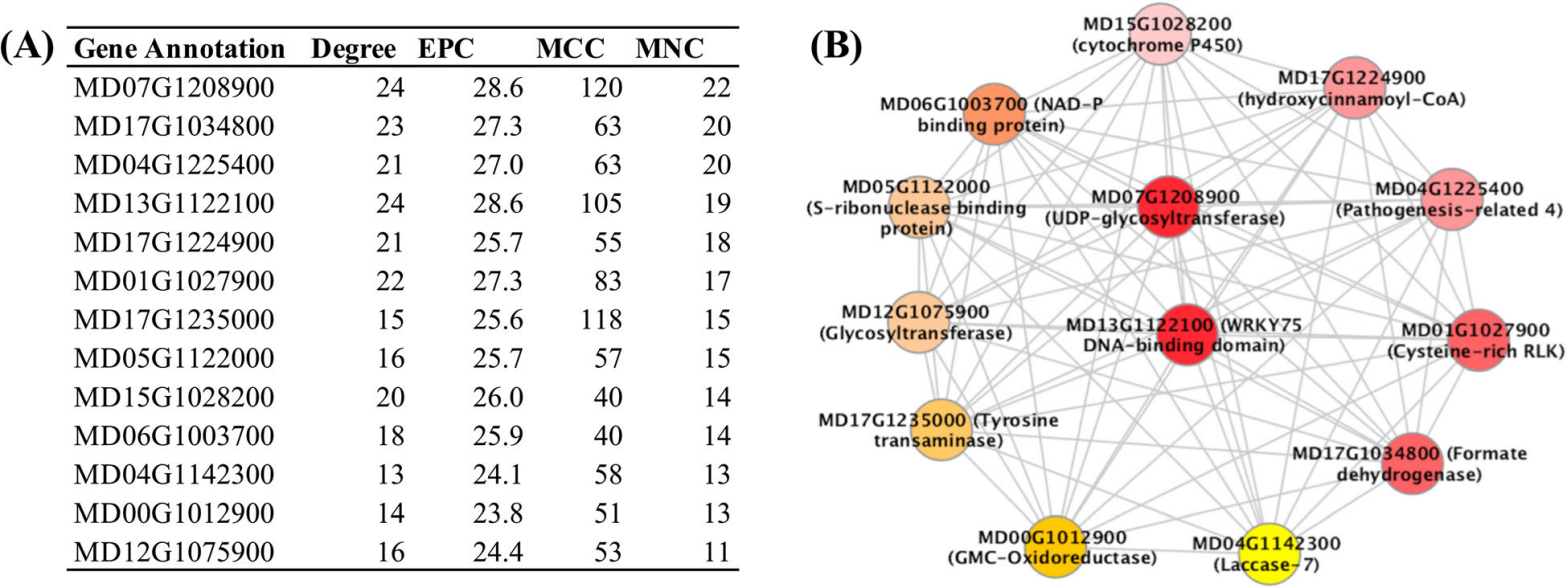
Hub genes identified from weighted gene co-expression network analysis of differentially expressed genes (DEGs). (**a**) Detected hub genes and their corresponding network connectivity scores as measured by degree, EPC, MCC, and MNC algorithms, Chin et al. (2014). (**b**) Interconnected sub-module of these hub genes, wherein different colors represent connectivities from highest (red) to lowest (yellow)

Subsequently, weighted co-expression analysis was used to identify co-expression patterns and putative interactions among RRFBR genes by identifying those hubs with the highest intramodular connectivity (Fig. 5a). It was found that a singular co-expression module “C3” represented 77.3% of RRFBR genes (Additional file 8: Figure S8). In addition, UDP-glycosyltransferase, formate dehydrogenase, pathogenesis-related 4, WRKY DNA-binding protein 75, cysteine-rich RLK, cytochrome P450, laccase 7, glucose-methanol-choline oxidoreductase protein, and glycosyltransferase family proteins were found to be consistently present as highly connected genes in the “C3” module (Fig. 5b; Additional file 18). This suggested that interactions among core genes from the general stress response, carbohydrate metabolism, and defense pathways determined observed differences in fire blight susceptibility between RAC-1 and RAC-4. In addition, detection of DNA-binding domain proteins as hubs in the “C3” module supported transcriptional regulation of co-expressed genes from these different pathways.

## Discussion

Earlier studies have reported that rootstocks and rootstock system architecture influence various important traits of scion genotypes grafted onto these rootstocks [20, 21]. As it has been demonstrated that rootstocks confer enhanced tolerance to salinity, drought, and disease in various crops [21–23], efforts have been undertaken to develop resistant rootstocks, which in turn can enhance disease tolerance of grafted scion cultivars [11, 24, 25]. In this study, it is observed that root traits of an apple rootstock (M.7), including root dry mass (g) and average roots per node (count), are indeed variable, and they do in turn influence shoot and leaf traits, including leaf chlorophyll contents, of different scion genotypes grafted onto this rootstock. More importantly, these root traits also influence response reactions of leaf and shoots of different scion genotypes to controlled inoculations with *E. amylovora*, and their susceptibility to fire blight disease. Although this latter finding confirms earlier reports [24, 26], it provides detailed analysis of the importance of root mass traits on fire blight reactions of grafted scion genotypes upon infection by *E. amylovora*. This finding is further supported by significant (*p < 0.05*) negative correlations obtained between root mass and scion fire blight susceptibility in these grafted apple trees.

Upon analysis of RACs of non-grafted ‘M.7’ rootstocks on fire blight susceptibility of above-ground leaf tissues, it is observed that increased root surface area contributed to decreased fire blight susceptibility in these above-ground leaf tissues, and the reverse is found to be true as well. These observed root-dependent differences in levels of fire blight disease susceptibility may be attributed to presence of multiple defense mechanisms [11, 25, 38–40] that interact with the pathways related to nutrient status of a plant. Indeed, involvement of diverse molecular pathways related to plant metabolism, cell cycle, oxidation-reduction, and stress response suggests presence of systemic regulation of fire blight infection in apples [11, 38]. Moreover, rootstock genotypes can significantly contribute to scion tolerance to fire blight susceptibility via rootstock-regulated gene expression patterns [11].

It is important to note that different fire blight disease reactions and root traits have displayed significant (*p* < 0.05) levels of variations in the two experiments conducted in this study. The observed variation in percent lesion length (%) could be attributed, in part, to the different genetic backgrounds of grafted scion cultivars, which was supported by calculated moderate broad-sense heritabilities of percent lesion length (%). The grafted scions could also influence the root traits of ‘M.7’ rootstock, but the short time frame (~ 3 months since grafting and 8 days post inoculation) of each experiment in this study makes it harder to notice such effects. However, the effect of scion genotypes cannot be fully excluded and could vary depending on the scion genotypes used. Nonetheless, the significant differences in root traits remains apparent in three different disease severity classes of mixed genetic backgrounds, which supports the observed relationships between root and disease severity traits. It has been reported that variations in root traits of rootstocks might contribute to phenotypic plasticity due to their exposure to different nutrient regimes, soil, and environmental conditions during their earlier growth in the clonal rootstock [15, 41, 42]. Phenotypic plasticity of different traits can vary among different genotypes [43–46]. Thus, identifying genes for root phenotypic plasticity would support efforts to breed for rootstocks with more uniform root traits. This, in turn, would contribute to enhanced resistance against *E. amylovora* infection, particularly for young grafted apple trees grown in orchards.

In this study, detection of various DEGs, including several disease-related and pathogenesis proteins, between contrasting RACs point to the critical role of the central immune system in conferring root-dependent fire blight susceptibility/resistance reactions. Earlier studies have reported that some of the disease-related CC-NBS-LRR proteins confer major resistance against fire blight in apples [39, 40]. These results suggest that gene interactions between core defense pathways and system-level metabolic and stress-responsive pathways may regulate root-dependent fire blight susceptibility/resistance reactions in apple. Moreover, these pathways may operate in coordination with sugar and carbohydrate metabolic pathways, which have demonstrated overrepresentation in contrasting RACs investigated in this study. Thus, it is likely that low root mass alters sink activities of a plant, which in turn can modify expression patterns of carbohydrate metabolism genes. Furthermore, changes in carbohydrate metabolism can alter a plant’s defense response through an inter-connected signaling network of metabolic and stress-responsive genes. For example, restriction of below-ground root growth can alter both development and carbohydrate metabolism of above-ground leaf tissues [47–49]. In addition, alteration in metabolite levels in a source leaf can determine the defense response against pathogen infection [50–52]. In fact, detection of a single co-expression module, “C3”, consisting of carbohydrate metabolism and disease-related proteins further supports viability of such a model in apple. Furthermore, presence of interacting WRKY and ethylene responsive DNA-binding transcription factors suggest transcriptional co-regulation of these pathways. Thus, low root mass may lead to resource-limiting conditions in the plant, thereby contributing to changes in gene expression of pathogenesis and disease-related proteins through carbohydrate metabolism pathways. It is these changes in expression in the central plant immune system that would then eventually determine fire blight susceptibility levels in apple. These results may add to the evidence that plants initiate a systemic response against fire blight after sensing the disease infection in inoculated scions, and transmit signals to the rootstock, which in turn contributes to disease tolerance/resistance through multiple mechanisms. However, rootstocks can influence the disease severity in grafted scions through multiple paths. For instance, rootstock-regulated gene expression, mobile RNA signaling between root-shoot, and nutrient-pathogen interaction as proposed in this study could be some of the factors contributing towards rootstock effects on grafted scions [4, 11, 25, 31].

It is important to point out that co-expression analysis conducted in this study has also highlighted those core genes with the highest intra-modular connectivity. In particular, the WRKY75 transcription factor and an UDP-glycotransferase are the top two genes displaying highest levels of connectivity within this network. Thus, these two genes are deemed as worthy candidates for further studies to assess their potential roles in various biotic and abiotic stress conditions. Furthermore, it will be interesting to identify those factors contributing to root-dependent differences in levels of fire blight susceptibility/resistance, which requires a rigorous experimental and functional validation of few candidate genes and is yet beyond the scope of this manuscript.

The root system directly regulates amounts of nutrients and water uptake, which in turn influence growth, physiology, and metabolism of grafted scions [22, 33, 53]. As smaller root systems can limit availability of nutrients or impose partial stress conditions, this in turn can influence levels of disease susceptibility. For instance, water deficit is reported to increase plant susceptibility against fungal infections in different plant species [54, 55]. This is partially attributed to altered expression of host R genes and/or of pathogen effectors [54]. In this study, contrasting expression patterns of several disease resistance genes and leucine rich repeats have been detected between RAC-1 and RAC-4, thus suggesting incidence of changes in plant immunity under low root surface areas. Similarly, nitrogen availability can affect disease severity levels in plants [53, 56]; however, the precise mechanism of nutrient-dependent changes in disease susceptibility levels remains unknown. Therefore, further studies should be conducted to determine the role(s) of resource limiting conditions and those factors involved in differences in root-dependent responses to fire blight susceptibility/resistance reactions in above-ground plant tissues.

Future studies are also needed to obtain more accurate estimates and interpretation of the relationships between root traits and disease severity as high variation in disease severity between replicates of the same genotype may have introduced noise in our analyses. Since several factors can contribute towards the high variation within genotypes, selection of uniform shoots at same age and equal amount of bacterial inoculum is one simple way to reduce this variation to some extent. In addition, using an increased number of biological replicates might also help to lower such variation within genotypes. Another informative set of experiments will be to extend similar analyses beyond the experimental time frame of this study as the results from this study suggest that disease progresses at different rates in plants with low and high root surface area.

## Conclusions

In summary, root traits can influence levels of fire blight susceptibility of apples. An optimum root area threshold is required to achieve the maximum tolerance against fire blight; however, high plasticity of root traits can hinder maintenance of such an optimal root system in apple rootstocks. Therefore, further studies should be conducted to identify genes or growth conditions that control root size, branching, and root phenotypic plasticity, as this new knowledge will assist in efforts to design more uniform root systems for optimum vigor of clonal apple rootstocks. In addition, manipulation of core regulatory genes of stress-responsive pathways can contribute to enhanced plant tolerance to abiotic and biotic stresses imposed by restricted root growth and disease infection.

## Methods

### Plant material and growth conditions

One-year-old apple rootstocks of ‘Malling 7’ (‘M.7’), a moderately fire blight-susceptible rootstock, were purchased from Willamette Nurseries Inc. (Canby, OR), and used in two different experiments. ‘M.7,’ a commercially important apple rootstock, was originally selected from traditional French rootstocks, known as ‘Doucin’, at East Malling Research Station (UK).

To evaluate influence of root mass of rootstocks on fire blight susceptibility of grafted scions, 45 different scion genotypes were grafted onto 1-year-old ‘M.7’ rootstocks (Additional file 1: Table S1). Bud-wood of scion genotypes was collected from the US National Apple Collection that is maintained from a long time at USDA-ARS Plant Genetic Resources Unit (PGRU) located in Geneva, NY. These grafted trees were maintained in a moist dark chamber for a period of three months to promote healing of the graft unions. Then, these grafted trees were planted in D40H deepots (Stuewe and Sons, Tangent, OR), 6.5 cm in diameter and 24.2 cm in depth, containing a standard Cornell soil mix (50 peatmoss:50 vermiculite with 6.2 kg.m^− 3^ lime, 1.25 kg.m^− 3^ superphosphate, and 0.62 kg.m^3^ calcium nitrate). These trees were allowed to acclimatize and grow in a greenhouse facility at Cornell AgriTech (Geneva, NY) maintained at 25 °C, 50% RH, and 16 h light/8 h dark photoperiod for a period of 8 weeks. For each scion genotype, three replications were maintained in the greenhouse facility at the Cornell AgriTech (Geneva, NY), and arranged in a completely randomized block design.

To assess the effects of varying root mass (g) on fire blight susceptibility, 21 non-grafted ‘M.7’ rootstocks were used in a second experiment. One-year-1-year-old ‘M.7’ rootstocks were pruned from the bottom up, using Fiskars hedge shears, to alter the numbers of adventitious root nodes growing along each of the rootstocks. The resulting rootstocks were photo-imaged, and analyzed using ImageJ (https://imagej.nih.gov/ij/) to identify four classes (RACs) that exhibit significantly different root area from one another. These non-grafted ‘M.7’ rootstocks were then potted in plastic pots (26 cm in diameter and 22.5 cm in depth) using the Cornell Soil mix as described above. For each RAC rootstock treatment, three replications were used, and these trees were maintained in the greenhouse facility at Cornell AgriTech, arranged in a completely randomized block design, under conditions of 25 °C, 50% RH, and 16 h light/8 h dark photoperiod for a period of 106 days.

### Fire blight inoculation and trait evaluation

Bacterial inoculum was prepared using a highly virulent *E. amylovora* strain, Ea2002A obtained from Dr. Steve Beer’s collection at Cornell University. Frozen inoculum stock was transferred to a petri plate containing King’s B medium (KB), and incubated for 48 h at 28 °C. Bacterial cells were recovered in a suspension culture using 1X PBS, and adjusted to a concentration of 10^9^ CFU/ml on a SmartSpec Plus Spectrophotometer (Bio-Rad Laboratories, Hercules, CA, USA).

Potted young trees were inoculated with either bacteria (treatment) or water (control; only in second experiment) for fire blight evaluation. The youngest unfolded leaf of an actively growing shoot of a potted young tree was inoculated by bisecting across the midribs using scissors dipped in the bacterial suspension, as described earlier [38, 57]. Deionized water was used to bisect midribs of leaves of control plants in the second experiment.

All inoculated young trees were evaluated 8 days after inoculation (dai) for fire blight infection, and for root traits. For both experiments, total shoot length, total leaf length, and length of necrosis of a leaf were measured in ‘cm’ using a ruler. The percent leaf lesion length (%) was calculated as the ratio of necrotic lesion length of a leaf to total leaf length multiplied by 100. Furthermore, chlorophyll contents of control and infected leaves were measured using a SPAD 502 Plus Chlorophyll Meter (Spectrum Technologies, Aurora, IL, USA). Average roots per node (count) and root dry mass (g) were simultaneously evaluated for all inoculated young trees (control and fire blight treated). Average roots per node were calculated by dividing total number of roots by number of nodes of a rootstock cutting used. At the end of each experiment at 8 dai, roots were shaved off each of the rootstocks, and dried in an oven to determine dry root mass.

For the second experiment, additional root trait data were digitally collected both at the beginning and at the end of the experiment. The root system from each ‘M.7’ rootstock was photographed by rotating it 360 degrees to capture the three-dimensional root surface area using a Canon EOS Rebel T5 Digital SLR camera (Cannon USA Inc., Melville, NY, USA). All raw images were first converted into greyscales, and then followed by binary conversion using the software ImageJ (https://imagej.nih.gov/ij/). Binary images were used to calculate the total root surface area (cm^2^) at the beginning of the experiment. Rootstocks were categorized into four different RACs, from lowest to highest root surface area (cm^2^). At the end of the experiment, roots were carefully dug out, and washed using a detergent and water. Roots were then spread on a flat surface, and photographed using a Canon EOS Rebel T5 Digital SLR camera. Photo-images were processed using an ImageJ software to calculate pre- and post-experiment root surface areas (cm^2^). Based on digital root diameter classifications, the root system of each young tree was separated into coarse (diameter > 1 mm) and fine (diameter < 1 mm) roots, dried in an oven, and then used to determine fine root dry mass (g), coarse root dry mass (g), and total root dry mass (g).

### Statistical analysis

All data collected for root and shoot traits, as well as for fire blight disease severity were used for statistical analysis. To test the relationships between root dry mass (g) and percent lesion length (%), the genotypes were grouped into three categories based on percent lesion length as Resistant (0–20% average PLL), Intermediate (21–80% average PLL), and Susceptible (81–100% average PLL). The data was tested for normality using Shapiro-Wilk test in R statistical software (http://www.R-project.org/) and log transformation was used to normalize the non-normal data. The normalized data was subjected to analysis of variance (ANOVA) using an R statistical software (http://www.R-project.org/). In addition, we also performed Kruskal-Wallis test using the original non-normal dataset to observe the significant differences. Mean values were compared using Tukey’s multiple comparison test. Broad-sense heritability (H^2^) was estimated as ratio of V_G_/V_P_, where V_P_ corresponded to the total phenotypic variance explained by the genetic component variance (V_G_). The absolute rate of disease progression was calculated as the difference in PLL between day 8 and day 2, divided by PLL at day 2.

Average trait values were used to calculate Pearson correlation coefficients, as well as to perform hierarchical clustering with the “hclust” function and a principal component analysis (PCA) using “prcomp” function in R (http://www.R-project.org/). Hierarchical clustering estimated individual relationships based on extent of similarities between them; whereas, PCA utilized variance components to determine such relationships. Trait mean values were scaled to conduct both hierarchical clustering and PCA analysis. For hierarchical clustering, scaled trait datasets were used to generate a Euclidean distance matrix for estimation of inter-cluster distance with Ward’s linkage method. PCA analysis was conducted to obtain principal component (PC) eigenvalues and rotations to estimate contributions of different traits to explain variation by each PC. A PCA biplot was generated using the first two principal components (PC1 and PC2) to determine the overall genotypic variation and effects of root dry mass (g) on disease severity.

### Leaf sample harvesting, RNA extraction, 3’RNAseq assay and sequencing

Leaf tissues from M.7 rootstocks of contrasting initial root surface areas (cm^2^), in the second experiment, were used for RNA extraction and for gene expression analysis. Leaves were collected at 4 and 8 dai from two biological replicates of control and three biological replicates of bacterial-inoculated young trees of two contrasting RACs as described earlier [38, 57]. Leaf tissues were immediately immersed in liquid nitrogen, and stored at -80 °C until used for RNA extraction.

A SpectrumTM Plant Total RNA Kit (Sigma-Aldrich, St. Louis, MO, USA) was used to extract total RNA as per manufacturer’s protocol. Leaf tissues were ground into fine powder in liquid nitrogen, and 100 mg of leaf powder was transferred to 500 μl of lysis solution containing 2% β-mercaptaethanol. Samples were thoroughly mixed, placed at 56 °C for 5 min, and centrifuged for 1 min at 13,000 rpm. The supernatant was passed through a filtration column at 13,000 rpm for 1 min to remove debris. The cleared lysate was mixed with 250 μl binding solution, and centrifuged through a binding column for 1 min at 13,000 rpm. After RNA binding, samples were washed twice using 500 μl wash solution I, as per manufacturer’s recommendations. Columns were centrifuged at maximum speed for 1 min during various washing steps. Dry columns were transferred to a new 2 ml centrifuge tube, and 50 μl elution buffer was added into the center of each binding column. Samples were kept in an elution buffer for 1 min, centrifuged at a maximum speed for 30 s to elute RNA, and then this was repeated using 30 μl of elution buffer to increase RNA yield. The amount of RNA was determined using a NanoDrop™ Spectrophotometer (Thermo Fisher Scientific, Grand Island, NY, USA), and the quality of RNA samples was assessed by running a 1% bleach agarose gel.

A total of 20 libraries were constructed and sequenced for RNA samples from control and bacterial-inoculated samples at the Genomics Facility at Cornell University (Ithaca, NY, USA). Briefly, 3’RNAseq libraries were prepared from ~ 500 ng of total RNA per sample using the Lexogen QuantSeq 3′ mRNA-Seq Library Prep Kit FWD for Illumina (https://www.lexogen.com/quantseq-3mrna-sequencing/). Libraries were quantified on a Molecular Devices Spectra Max M2 plate reader (with the intercalating dye QuantiFluor), and pooled accordingly for maximum evenness. The pooled sample was quantified by digital PCR, and sequenced along a single lane of an Illumina NextSeq500 sequencer to obtain single-end 1 × 86 bp sequences. Pooled libraries were de-multiplexed based upon six-base i7 indices using an Illumina bcl2fastq2 software (version 2.17; Illumina, Inc., San Diego, CA, USA).

### Sequencing data processing and analysis

A Trimmomatic (version 0.36) [58] software was used to remove Illumina adapters from de-multiplexed fastq sequences, as well as to remove low-quality reads for further analysis. Poly-A tails and poly-G stretches of at least 10 bases in length were then removed using the BBDuk program in the package BBMap (https://sourceforge.net/projects/bbmap/), but keeping reads of at least 18 bases in length after trimming. Often, poly-G stretches are obtained from sequencing past ends of short fragments (G = no signal).

Trimmed reads were then aligned to the GDDH13 Version 1.1 apple genome assembly (https://iris.angers.inra.fr/gddh13/downloads/GDDH13_1-1_formatted.fasta.bz2) using the STAR aligner (version 2.5.3a) [59]. For the STAR indexing step, the gff3 annotation file (https://iris.angers.inra.fr/gddh13/downloads/gene_models_20170612.gff3.bz2) was converted into a gtf format the gffread program from cufflinks (version 2.2.1) [60]. Key parameters used in the STAR indexing step (−-runMode genomeGenerate) include --genomeChrBinNbits 18 and --sjdbOverhang 100. The STAR alignment step used the following key parameters: --outReadsUnmapped Fastx, −-outFilterMultimapNmax 10, −-outFilterMismatchNoverLmax 0.06, −-outSAMmode Full, −-outSAMattributes Standard, −-outFilterIntronMotifs, and RemoveNoncanonicalUnannotated. Output SAM files were converted to BAM using SAMtools (version 1.6) [61], and numbers of reads overlapping each gene in the gff3 file along the forward strand were counted using a HTSeq-count (version 0.6.1) [62]. A gene was deemed to be expressed using a criterion of detecting a minimum of five aligned high-quality read sequences against a particular gene model.

### Gene expression and enrichment analysis

The R package DESeq2 (version 1.20.0) [63] was used to obtain normalized counts from raw read counts. These counts were then used to conduct PCA of the 500 most variably-expressed genes following count normalization and variance stabilizing transformation, as well as for differential gene expression analysis. Control and bacterial-inoculated samples of each root class and time points were compared by deeming root class, time point, and inoculation treatment as distinct factors. The “contrast” function in DESeq2 was used to obtain expression analysis output for each comparison. For each gene, statistical significance of differential expression was based on a Wald test for a non-zero log fold change (LFC) estimate obtained from fitting a negative binomial generalized linear model [63]. Adjustment of *p*-values for multiple testing followed the Benjamini and Hochberg method [64]. Genes were deemed differentially expressed based on a log2Fold change threshold of 1.5 and a *p*-value of less than 0.01. All upregulated genes were determined based on positive log2Fold change values, and vice-versa.

Sets of differentially expressed genes from individual comparisons were used to perform a gene ontology (GO) term enrichment analysis using Fisher’s exact test with agriGO v2.0 [65]. Differentially expressed genes (DEGs) were compared to the complete set of fully-annotated genes in the GDDH13 Version 1.1 apple genome assembly. Estimated *p*-values were corrected using the Hochberg false discovery rate (FDR) correction method in agriGO v2.0. A *p*-value cutoff of less than 0.05 was used to determine significantly enriched GO terms.

### Gene co-expression network analysis

A co-expression network analysis of DE genes was performed using the weighted gene co-expression network analysis (WGCNA) package in R [66] to obtain modules of genes having similar expression patterns. A list of unique DE genes from each comparison was established by removing redundant genes from various differential gene expression analyses. Subsequently, normalized gene expression values for these unique DEGs were extracted to perform a co-expression network analysis. Co-expressed gene modules were then built using a single-step network generation and a module detection approach in WGCNA. Specifically, a network was generated by connecting all expression values in a dataset followed by detection of modules exhibiting very similar patterns of gene expression. A threshold value for module assignments was estimated using an unsigned topological overlap matrix (TOM); whereas, networks were constructed using a threshold power of 16, branch cut height of 0.25, and a minimum module size of 30. These co-expression modules were then visualized in Cytoscape v3.7.1 [67], and those highly connected genes, within each module, were identified using cytoHubba plugin [68] in Cytoscape. These modules were exported from WGCNA using a threshold of 0.2, and the top 20 highly connected genes were identified using four different algorithms including MCC, MNC, Degree, and EPC, implemented in cytoHubba. Finally, outputs were compared to select those hub genes that were consistently detected from all the four algorithms.

## Supplementary information

**Additional file 1: Figure S1.** Boxplots showing variation in different root (A-B), shoot (C-E), and fire blight infection (F) traits in 45 grafted scion genotypes on ‘M.7’ rootstocks.

**Additional file 2: Figure S2.** Pearson correlation coefficients (R^2^) of root dry mass (g) against percent lesion length (%) of 45 grafted scion genotypes on ‘M.7’ rootstocks.

**Additional file 3: Figure S3.** Hierarchical genotype clustering of 45 grafted scion genotypes on ‘M.7’ rootstocks and cluster mean heatmap for different traits.

**Additional file 4: Figure S4.** Barplot showing rotations of first three principal components for different traits.

**Additional file 5: Figure S5.** Disease progression (percent lesion length) over time (2, 4, 6 and 8 dai).

**Additional file 6: Figure S6.** Volcano plots showing differentially expressed genes (DEGs) at 4 dai (A), and 8 dai (B). The unique and common DEGs are also shown in Venn Diagram (C).

**Additional file 7: Figure S7.** Heat map showing expression patterns of DEGs in protein kinase, carbohydrate metabolism, and defense pathways.

**Additional file 8: Figure S8.** Heat map showing expression patterns in three co-expression modules detected from weighted gene co-expression analysis.

**Additional file 9: Table S1.** List of genotypes used for analysis of relationships between root traits and disease severity.

**Additional file 10: Table S2.** Pairwise correlation coefficients between root, shoot, and disease traits in 45 grafted scions on ‘M.7’ rootstocks.

**Additional file 11: Table S3.** Pairwise correlation coefficients between root, shoot, and disease traits in different root area classes (RACs).

**Additional file 12: Table S4.** Summary of sequencing and read alignments against the apple genome assembly.

**Additional file 13.** Principal component analysis (PCA) using normalized read counts of apple genes.

**Additional file 14.** DEGs between RAC-1 and RAC-4 from control samples at 4 and 8 dai.

**Additional file 15.** DEGs between control and fire blight infected samples in RAC-1 and RAC-4 at 4 and 8 dai.

**Additional file 16.** Functional annotations and expression patterns of common 454 DEGs.

**Additional file 17.** Pathways showing enrichment in the common 454 DEGs.

**Additional file 18.** Hub genes in the “C3” co-expression module.

## Data Availability

All read sequences from RNA-Seq analysis for all samples used in this study have been deposited in the National Center for Biotechnology Information (NCBI) sequence read archive (SRA) database under the Bioproject identifier PRJNA507638. The data supporting the conclusions of this research is provided as supplementary information.

## Abbreviations

ANOVA: Analysis of Variance
BAM: Binary Alignment Map
CA: California
CC-NBS-LRR: Coiled Coil Nucleotide Binding Site Leucine Rich Repeats
CFU: Colony-forming Unit
DEG: Differentially Expressed Gene
EPC: Edge Percolated Component
FDR: False Discovery Rate
GDDH: Golden Delicious Double Haploid
GO: Gene Ontology
IL: Illinois
KB: King’s B medium
LFC: Log Fold Change
M.7: Malling 7
MCC: Maximal Clique Centrality
MNC: Maximum Neighborhood Component
NCBI: National Center for Biotechnology Information
NY: New York
OR: Oregon
PBS: Phosphate-buffered Saline
PC: Principal Component
PCA: Principal Component Analysis
PCR: Polymerase Chain Reaction
PGRU: Plant Genetic Resources Unit
PLL: Percent Lesion Length
RAC: Root Area Class
RH: Relative Humidity
RRFBR: Root-regulated Fire Blight Responsive
RSA: Root System Architecture
SAM: Sequence Alignment Map
SPAD: Soil Plant Analysis Development
SRA: Sequence Read Archive
STAR: Spliced Transcripts Alignment to a Reference
TOM: Topological Overlap Matrix
UK: United Kingdom
US: United States
WGCNA: Weighted Gene Co-expression Network Analysis
