# Supplementary figures and images for: Root system traits impact early fire blight susceptibility in apple (Malus × domestica)
Source: BMC Plant Biol. 2019 Dec 23;19:579. doi: 10.1186/s12870-019-2202-3 (PMC6929320; doi:10.1186/s12870-019-2202-3)

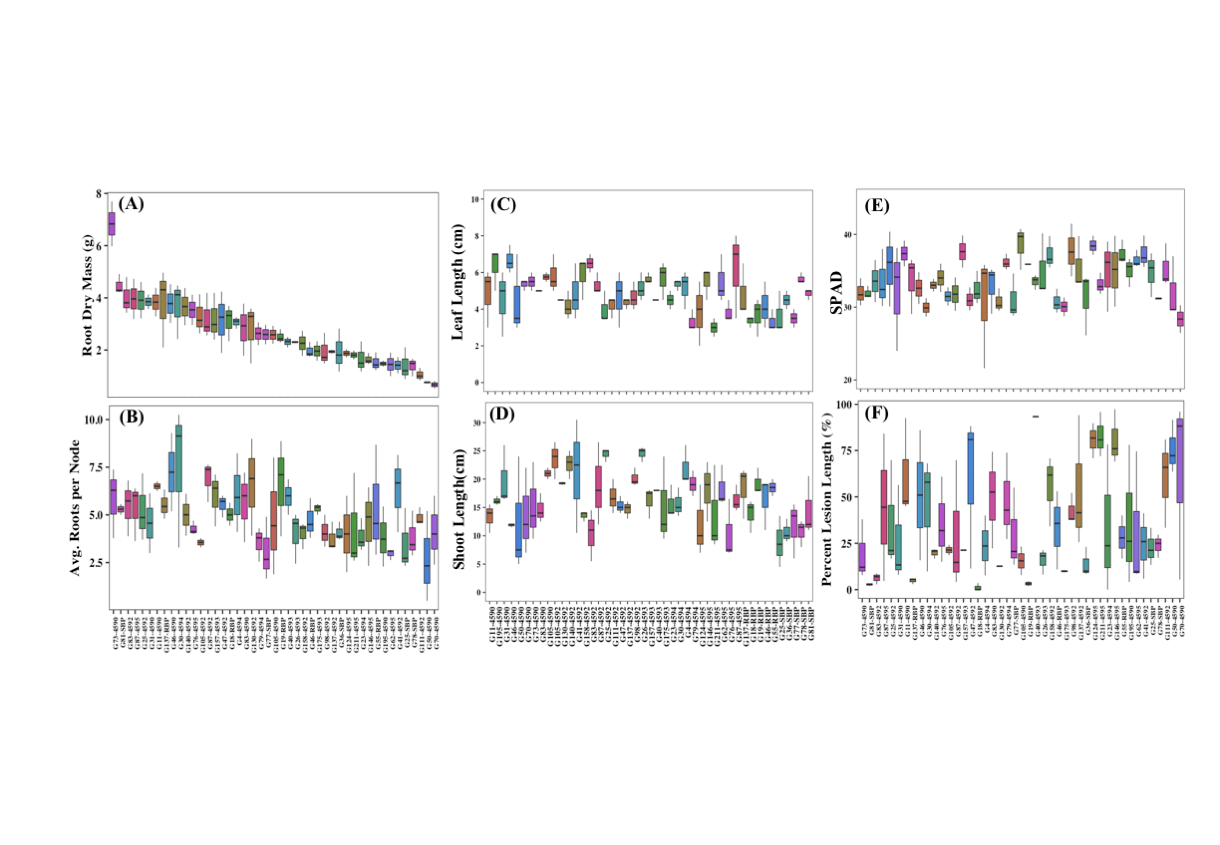

Supplement: Supplementary file 1 — Additional file 1: Figure S1. Boxplots showing variation in different root (A-B), shoot (C-E), and fire blight infection (F) traits in 45 grafted scion genotypes on ‘M.7’ rootstocks. [file 12870_2019_2202_MOESM1_ESM.tiff]

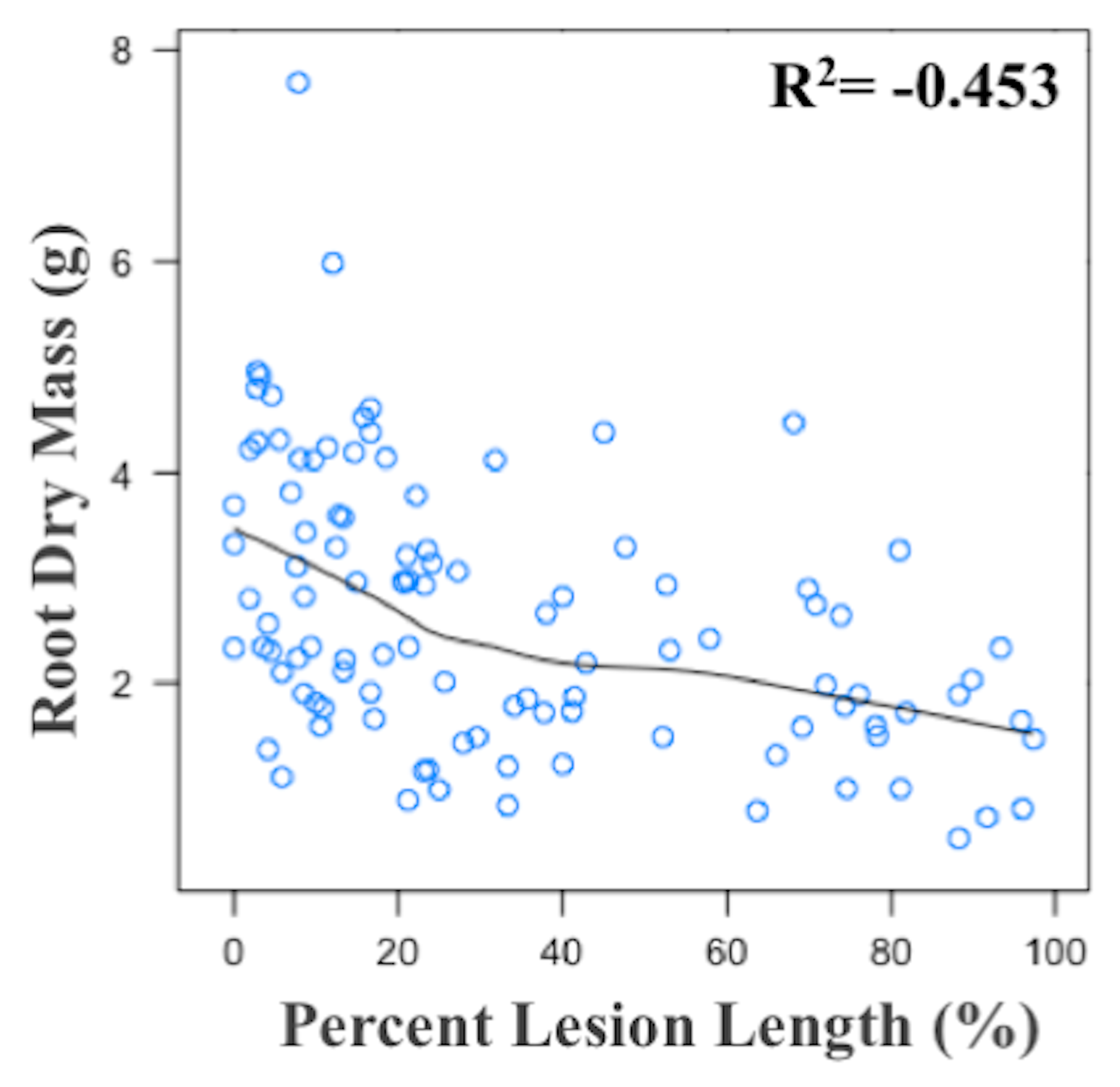

Supplement: Supplementary file 2 — Additional file 2: Figure S2. Pearson correlation coefficients (R2) of root dry mass (g) against percent lesion length (%) of 45 grafted scion genotypes on ‘M.7’ rootstocks. [file 12870_2019_2202_MOESM2_ESM.tiff]

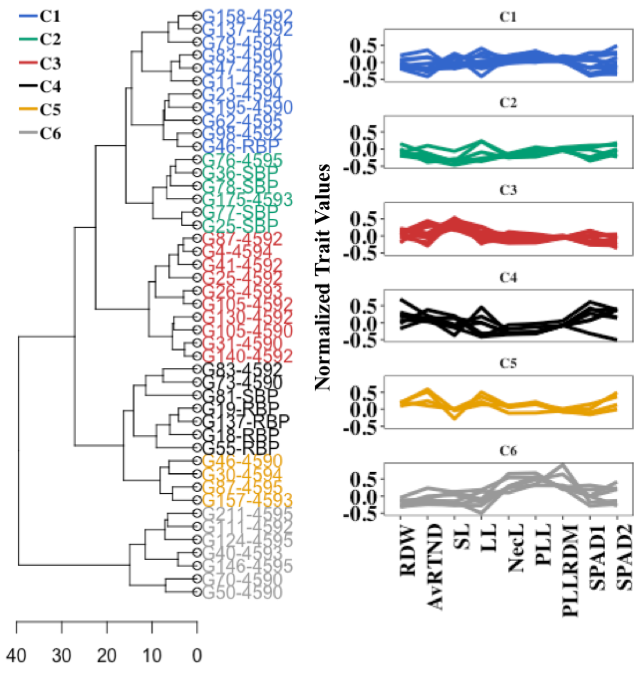

Supplement: Supplementary file 3 — Additional file 3: Figure S3. Hierarchical genotype clustering of 45 grafted scion genotypes on ‘M.7’ rootstocks and cluster mean heatmap for different traits. [file 12870_2019_2202_MOESM3_ESM.png]

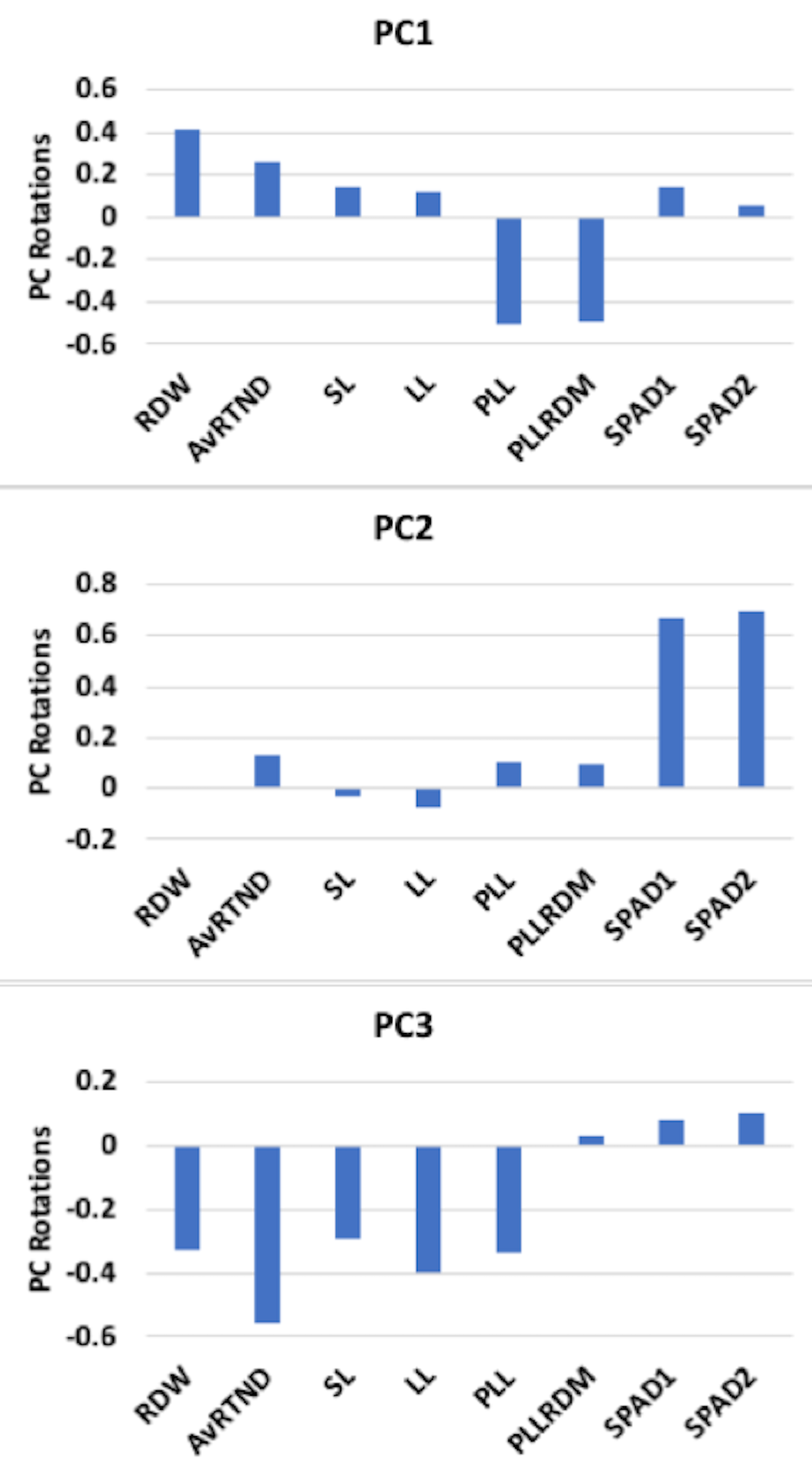

Supplement: Supplementary file 4 — Additional file 4: Figure S4. Barplot showing rotations of first three principal components for different traits. [file 12870_2019_2202_MOESM4_ESM.tiff]

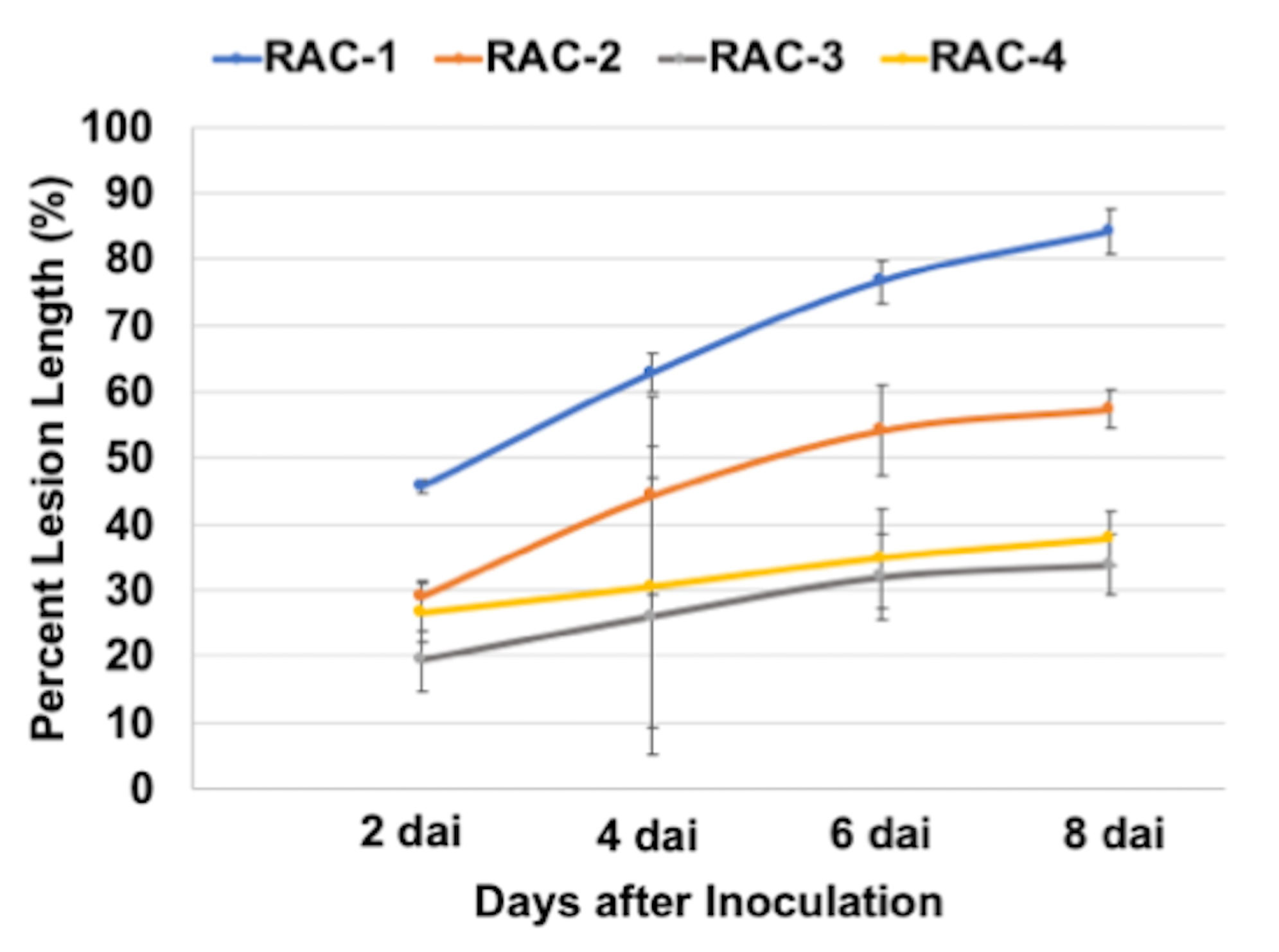

Supplement: Supplementary file 5 — Additional file 5: Figure S5. Disease progression (percent lesion length) over time (2, 4, 6 and 8 dai). [file 12870_2019_2202_MOESM5_ESM.png]

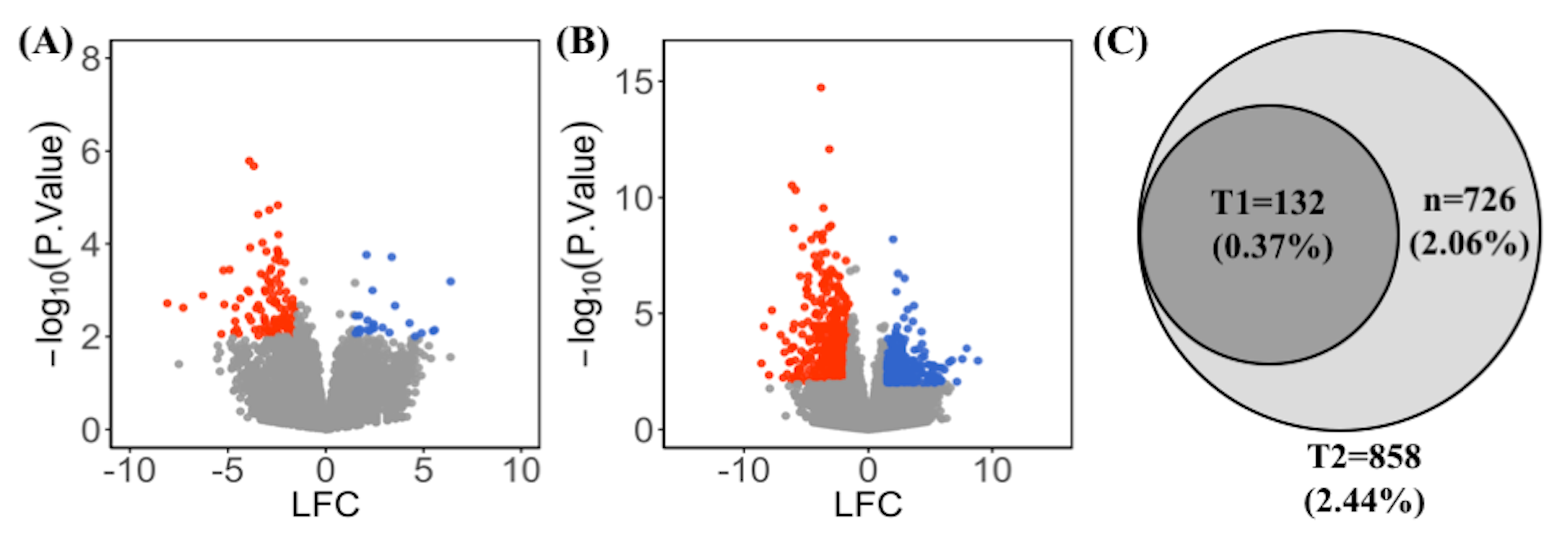

Supplement: Supplementary file 6 — Additional file 6: Figure S6. Volcano plots showing differentially expressed genes (DEGs) at 4 dai (A), and 8 dai (B). The unique and common DEGs are also shown in Venn Diagram (C). [file 12870_2019_2202_MOESM6_ESM.png]

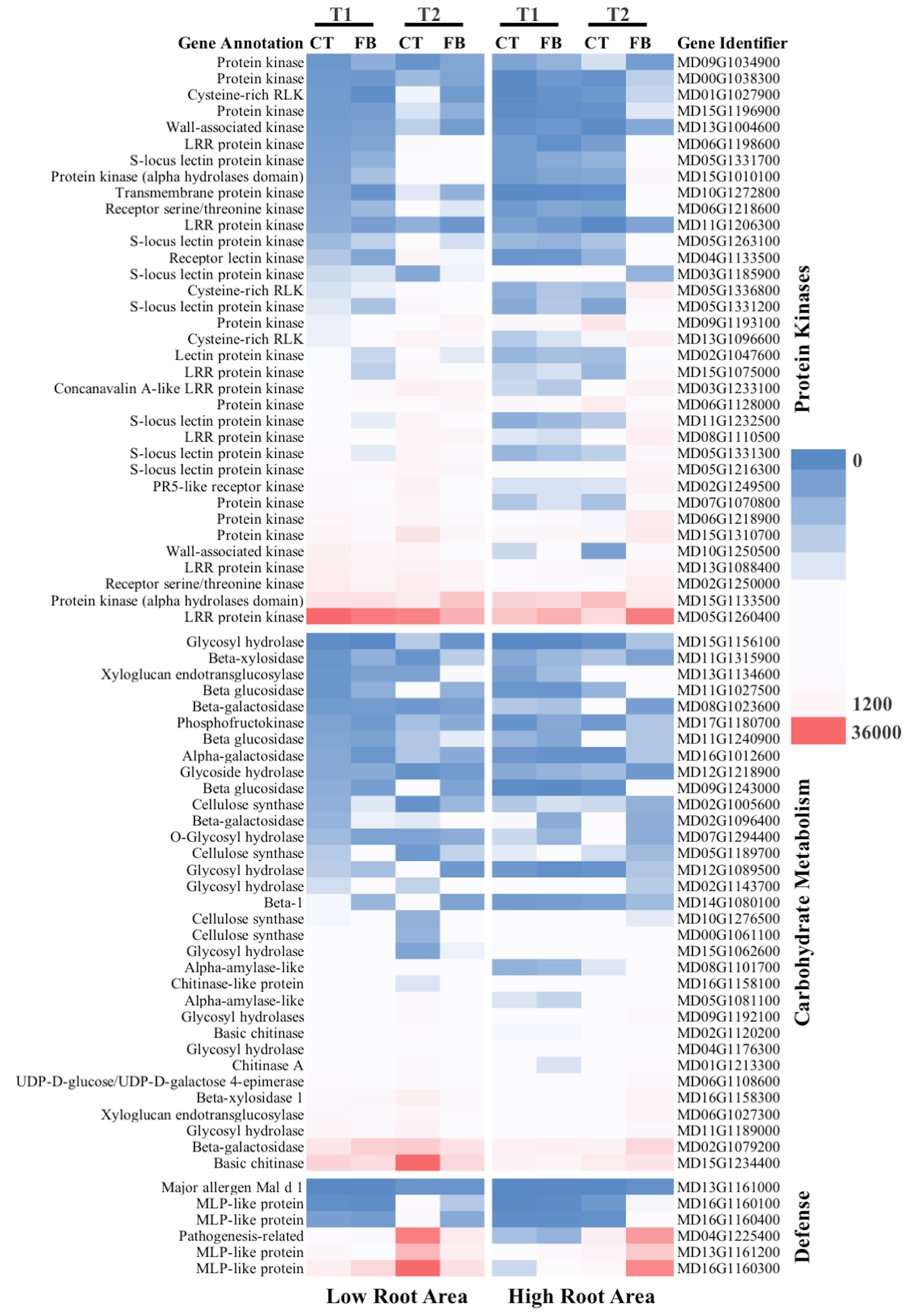

Supplement: Supplementary file 7 — Additional file 7: Figure S7. Heat map showing expression patterns of DEGs in protein kinase, carbohydrate metabolism, and defense pathways. [file 12870_2019_2202_MOESM7_ESM.png]

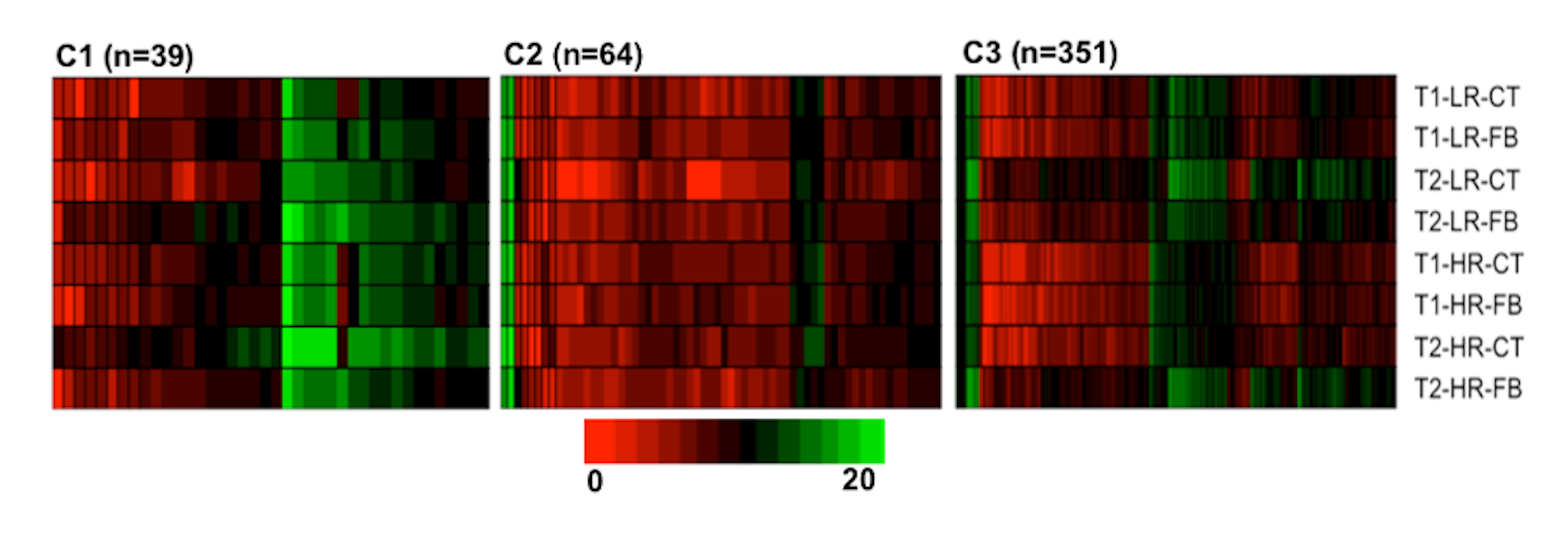

Supplement: Supplementary file 8 — Additional file 8: Figure S8. Heat map showing expression patterns in three co-expression modules detected from weighted gene co-expression analysis. [file 12870_2019_2202_MOESM8_ESM.png]
