# Supplementary material for: Root system traits impact early fire blight susceptibility in apple (Malus × domestica)
Source: BMC Plant Biol. 2019 Dec 23;19:579. doi: 10.1186/s12870-019-2202-3 (PMC6929320; doi:10.1186/s12870-019-2202-3)
Supplement: Supplementary file 9 — Additional file 9: Table S1. List of genotypes used for analysis of relationships between root traits and disease severity. [file 12870_2019_2202_MOESM9_ESM.docx]

**Supplementary Table S1**. List of genotypes used for analysis of relationships between root traits and disease severity

| **ID** | **Genotype** | **Population** | **PLL Class** | **PLL Standard Deviation** |
| --- | --- | --- | --- | --- |
| G105-4590 | G105 | 4590 | Resistant | Low |
| G105-4592 | G105 | 4592 | Intermediate | Low |
| G11-4590 | G11 | 4590 | Intermediate | Moderate-High |
| G111-4592 | G111 | 4592 | Intermediate | Moderate-High |
| G124-4595 | G124 | 4595 | Susceptible | Low |
| G130-4592 | G130 | 4592 | Resistant | Low |
| G137-4592 | G137 | 4592 | Intermediate | Moderate-High |
| G137-RBP | G137 | RBP | Resistant | Low |
| G140-4592 | G140 | 4592 | Resistant | Low |
| G146-4595 | G146 | 4595 | Susceptible | Low |
| G157-4593 | G157 | 4593 | Intermediate | Low |
| G158-4592 | G158 | 4592 | Intermediate | Moderate-High |
| G175-4593 | G175 | 4593 | Resistant | Low |
| G18-RBP | G18 | RBP | Resistant | Low |
| G19-RBP | G19 | RBP | Resistant | Low |
| G195-4590 | G195 | 4590 | Intermediate | Moderate-High |
| G211-4595 | G211 | 4595 | Susceptible | Low |
| G23-4594 | G23 | 4594 | Intermediate | Moderate-High |
| G25-4592 | G25 | 4592 | Intermediate | Moderate-High |
| G25-SBP | G25 | SBP | Intermediate | Low |
| G26-4593 | G26 | 4593 | Resistant | Low |
| G30-4594 | G30 | 4594 | Intermediate | Moderate-High |
| G31-4590 | G31 | 4590 | Intermediate | Moderate-High |
| G36-SBP | G36 | SBP | Resistant | Low |
| G4-4594 | G4 | 4594 | Intermediate | Low |
| G40-4593 | G40 | 4593 | Susceptible | Low |
| G41-4592 | G41 | 4592 | Intermediate | Low |
| G46-4590 | G46 | 4590 | Intermediate | Moderate-High |
| G46-RBP | G46 | RBP | Intermediate | Moderate-High |
| G47-4592 | G47 | 4592 | Intermediate | Moderate-High |
| G50-4590 | G50 | 4590 | Intermediate | Low |
| G55-RBP | G55 | RBP | Intermediate | Low |
| G62-4595 | G62 | 4595 | Intermediate | Moderate-High |
| G70-4590 | G70 | 4590 | Intermediate | Moderate-High |
| G73-4590 | G73 | 4590 | Resistant | Low |
| G76-4595 | G76 | 4595 | Intermediate | Moderate-High |
| G77-SBP | G77 | SBP | Intermediate | Moderate-High |
| G78-SBP | G78 | SBP | Intermediate | Low |
| G79-4594 | G79 | 4594 | Intermediate | Moderate-High |
| G81-SBP | G81 | SBP | Resistant | Low |
| G83-4590 | G83 | 4590 | Intermediate | Moderate-High |
| G83-4592 | G83 | 4592 | Resistant | Low |
| G87-4592 | G87 | 4592 | Intermediate | Moderate-High |
| G87-4595 | G87 | 4595 | Intermediate | Moderate-High |
| G98-4592 | G98 | 4592 | Intermediate | Low |
