# Supplementary material for: Root system traits impact early fire blight susceptibility in apple (Malus × domestica)
Source: BMC Plant Biol. 2019 Dec 23;19:579. doi: 10.1186/s12870-019-2202-3 (PMC6929320; doi:10.1186/s12870-019-2202-3)
Supplement: Supplementary file 10 — Additional file 10: Table S2. Pairwise correlation coefficients between root, shoot, and disease traits in 45 grafted scions on ‘M.7’ rootstocks. [file 12870_2019_2202_MOESM10_ESM.docx]

**Supplementary Table S2**. Pairwise Pearson correlation coefficients between different root growth and disease susceptibility traits in the population consisting of 45 grafted scions on M.7 rootstock. Lower triangle represents the correlation “r2” values. The * indicates a significant correlation at *p-value* threshold < 0.05.

|  | **Root Dry Mass** | **Avg. Roots per Node** | **Percent Lesion Length** | **SPAD** | **Shoot Length** | **Leaf Length** |
| --- | --- | --- | --- | --- | --- | --- |
| **Root Dry Mass** | 1.00 |  |  |  |  |  |
| **Avg. Roots per Node** | 0.27* | 1.00 |  |  |  |  |
| **Percent Lesion Length** | -0.45* | -0.12 | 1.00 |  |  |  |
| **SPAD** | 0.00 | -0.03 | 0.16 | 1.00 |  |  |
| **Shoot Length** | 0.14 | 0.09 | -0.17 | -0.20* | 1.00 |  |
| **Leaf Length** | 0.23* | 0.13 | -0.20* | 0.04 | -0.05 | 1.00 |
