# Supplementary material for: Root system traits impact early fire blight susceptibility in apple (Malus × domestica)
Source: BMC Plant Biol. 2019 Dec 23;19:579. doi: 10.1186/s12870-019-2202-3 (PMC6929320; doi:10.1186/s12870-019-2202-3)
Supplement: Supplementary file 11 — Additional file 11: Table S3. Pairwise correlation coefficients between root, shoot, and disease traits in different root area classes (RACs). [file 12870_2019_2202_MOESM11_ESM.docx]

**Supplementary Table S3**. Pairwise Pearson correlation coefficients between different root growth and disease susceptibility traits in the low to high root area classes (RACs). Lower triangle represents the correlation “r2” values. The * indicates a significant correlation at *p-value* threshold < 0.05.

|  | **Root Area Pre-Plant** | **Root Area Post-Plant** | **Avg. Roots per Node** | **Coarse Root Dry Mass** | **Fine Root Dry Mass** | **Total Root Dry Mass** | **Leaf Length** | **Shoot Length** | **Percent Lesion Length** | **SPAD** |
| --- | --- | --- | --- | --- | --- | --- | --- | --- | --- | --- |
| **Root Area Pre-Plant** | 1.00 |  |  |  |  |  |  |  |  |  |
| **Root Area Post-Plant** | 0.88* | 1.00 |  |  |  |  |  |  |  |  |
| **Avg. Roots per Node** | 0.67* | 0.56 | 1.00 |  |  |  |  |  |  |  |
| **Coarse Root Dry Mass** | 0.80* | 0.86* | 0.39 | 1.00 |  |  |  |  |  |  |
| **Fine Root Dry Mass** | 0.62* | 0.79* | 0.31 | 0.54 | 1.00 |  |  |  |  |  |
| **Total Root Dry Mass** | 0.83* | 0.94* | 0.43 | 0.96* | 0.76* | 1.00 |  |  |  |  |
| **Leaf Length** | -0.23 | 0.05 | -0.20 | 0.13 | 0.23 | 0.19 | 1.00 |  |  |  |
| **Shoot Length** | 0.78* | 0.71* | 0.40 | 0.56 | 0.78* | 0.68* | -0.33 | 1.00 |  |  |
| **Percent Lesion Length** | -0.70* | -0.54* | -0.35 | -0.45 | -0.58* | -0.53 | 0.32 | -0.82* | 1.00 |  |
| **SPAD** | -0.06 | 0.22 | -0.20 | 0.23 | 0.12 | 0.23 | 0.22 | 0.00 | 0.65 | 1.00 |
