# Supplementary material for: Root system traits impact early fire blight susceptibility in apple (Malus × domestica)
Source: BMC Plant Biol. 2019 Dec 23;19:579. doi: 10.1186/s12870-019-2202-3 (PMC6929320; doi:10.1186/s12870-019-2202-3)
Supplement: Supplementary file 12 — Additional file 12: Table S4. Summary of sequencing and read alignments against the apple genome assembly. [file 12870_2019_2202_MOESM12_ESM.docx]

**Supplementary Table S4.** Summary statistics of read sequences, quality filtration and read alignments to the apple genome assembly (GDDH13 v1.1). Here, “FB” represents fire blight.

| **Sample Identifier** | **Time** | **Root Area Class** | **Treatment** | **Total Reads** | **Mapped Reads** | **Mapped Read (%)** | **Uniquely Mapped Reads** | **Uniquely Mapped Reads (%)** | **Uniquely Mapped No Feature** | **Uniquely Mapped No Feature (%)** | **Uniquely Mapped Ambiguous** | **Uniquely Mapped Ambiguous (%)** |
| --- | --- | --- | --- | --- | --- | --- | --- | --- | --- | --- | --- | --- |
| A3 | 1 | Low Root Area | Control | 21180280 | 20119282 | 94.99 | 19534838 | 97.10 | 3744427 | 19.17 | 6139 | 0.031 |
| A7 | 1 | Low Root Area | Control | 23610852 | 22020813 | 93.27 | 21120159 | 95.91 | 4144002 | 19.62 | 7928 | 0.038 |
| A19 | 1 | Low Root Area | FB Infection | 11307574 | 10475633 | 92.64 | 9964333 | 95.12 | 2007132 | 20.14 | 3738 | 0.038 |
| A20 | 1 | Low Root Area | FB Infection | 31466810 | 29762293 | 94.58 | 28700119 | 96.43 | 4991064 | 17.39 | 10091 | 0.035 |
| A21 | 1 | Low Root Area | FB Infection | 36086647 | 33881101 | 93.89 | 32629381 | 96.31 | 5419734 | 16.61 | 11806 | 0.036 |
| A1 | 1 | High Root Area | Control | 20090173 | 19109649 | 95.12 | 18531091 | 96.97 | 3337110 | 18.01 | 6026 | 0.033 |
| A12 | 1 | High Root Area | Control | 19445233 | 17974338 | 92.44 | 17270679 | 96.09 | 3495098 | 20.24 | 6070 | 0.035 |
| A6 | 1 | High Root Area | FB Infection | 17665919 | 16427722 | 92.99 | 15900196 | 96.79 | 2927248 | 18.41 | 4647 | 0.029 |
| A10 | 1 | High Root Area | FB Infection | 25266421 | 23712152 | 93.85 | 22754543 | 95.96 | 4828468 | 21.22 | 8198 | 0.036 |
| A16 | 1 | High Root Area | FB Infection | 11935411 | 10956001 | 91.79 | 10492349 | 95.77 | 2523171 | 24.05 | 3444 | 0.033 |
| C3 | 2 | Low Root Area | Control | 29836822 | 27758085 | 93.03 | 26446214 | 95.27 | 4841231 | 18.31 | 10245 | 0.039 |
| C7 | 2 | Low Root Area | Control | 22932434 | 21004468 | 91.59 | 19880589 | 94.65 | 3541127 | 17.81 | 8291 | 0.042 |
| C19 | 2 | Low Root Area | FB Infection | 33192591 | 31222469 | 94.07 | 29938993 | 95.89 | 5046364 | 16.86 | 12679 | 0.042 |
| C20 | 2 | Low Root Area | FB Infection | 19921474 | 18808212 | 94.41 | 17738502 | 94.31 | 3039293 | 17.13 | 8065 | 0.045 |
| C21 | 2 | Low Root Area | FB Infection | 17507663 | 16351274 | 93.40 | 15438514 | 94.42 | 2719832 | 17.62 | 6131 | 0.040 |
| C1 | 2 | High Root Area | Control | 14039558 | 13326800 | 94.92 | 12713494 | 95.40 | 2093280 | 16.47 | 5243 | 0.041 |
| C12 | 2 | High Root Area | Control | 26200054 | 24378463 | 93.05 | 23110011 | 94.80 | 4465324 | 19.32 | 9804 | 0.042 |
| C6 | 2 | High Root Area | FB Infection | 23196883 | 21518725 | 92.77 | 20398820 | 94.80 | 4041522 | 19.81 | 7956 | 0.039 |
| C10 | 2 | High Root Area | FB Infection | 20231913 | 18798944 | 92.92 | 17839714 | 94.90 | 3386830 | 18.99 | 7411 | 0.042 |
| C16 | 2 | High Root Area | FB Infection | 29356803 | 27352597 | 93.17 | 26345505 | 96.32 | 5455078 | 20.71 | 9124 | 0.035 |
| **Average** |  |  |  | 22723576 | 21247951 | 93.44 | 20337402 | 95.66 | 3802367 | 18.89 | 7652 | 0.038 |
| **Sum** |  |  |  | 454,471,515 | 424959021 |  | 406748044 |  | 76047335 |  | 153036 |  |
